# From Stroke Suspicion to Genetic Confirmation: Familial Hemiplegic Migraine Type 2 as a Rare Stroke Mimic With Persistent Hemiplegia

**DOI:** 10.7759/cureus.93313

**Published:** 2025-09-26

**Authors:** Mohamed Haggag, Syed Hussaini, Ahmed Shehabeldein, Suzanne Muhammad

**Affiliations:** 1 Respiratory Medicine, Blackpool Teaching Hospitals NHS Foundation Trust, Blackpool, GBR; 2 Respiratory Medicine, Blackpool Victoria Hospital, Blackpool, GBR; 3 General Medicine, Blackpool Victoria Hospital, Blackpool, GBR; 4 Emergency Medicine, AlHayat Specialized Hospital, Port Said, EGY

**Keywords:** atp1a2 mutation, familial hemiplegic migraine type 2, genetic diagnosis, hemiplegic migraine, nimodipine, persistent hemiplegia, seizures, stroke mimic

## Abstract

Hemiplegic migraine (HM) is a rare migraine subtype characterized by reversible motor aura that can closely mimic acute ischemic stroke. Familial HM (FHM) is an autosomal dominant condition linked to mutations in CACNA1A, ATP1A2, and SCN1A genes. FHM type 2 (FHM2), associated with ATP1A2 mutations, disrupts astrocytic Na⁺/K⁺-ATPase function and may present with seizures, encephalopathy, or prolonged neurological deficits. Accurate recognition is critical, as misdiagnosis can result in inappropriate interventions.

We report the case of a 33-year-old woman who presented with a gradual onset of severe left-sided headache, dysphasia, and hemiparesis over a two-day period. Shortly after initial imaging, she developed tonic-clonic seizures, loss of consciousness, and pyrexia, requiring intubation and intensive care unit (ICU) admission. Non-contrast computed tomography (CT) of the brain excluded hemorrhage. Magnetic resonance imaging (MRI) demonstrated right parietal and temporal cortical diffusion restriction. CT angiography suggested right middle cerebral artery (MCA) occlusion, but digital subtraction angiography (DSA) confirmed vasospasm without thromboembolism. Electroencephalography revealed diffuse encephalopathy. A family history of recurrent transient hemiparesis was noted. Genetic testing with whole-exome sequencing (WES) confirmed an ATP1A2 mutation consistent with FHM2. The patient improved in consciousness and language after nimodipine therapy but had persistent hemiplegia at discharge.

This case illustrates the significant diagnostic overlap between HM and acute ischemic stroke. The combination of seizures, encephalopathy, fever, and diffusion restriction on MRI is atypical for FHM and can complicate diagnosis. Inappropriate thrombolysis or invasive interventions may result if stroke mimics are not considered. Previous reports of ATP1A2-associated FHM2 describe variable phenotypes, including seizures and prolonged neurological deficits. The use of nimodipine remains controversial, with both clinical improvement and deterioration reported in the literature. Genetic confirmation provides diagnostic clarity, informs prognosis, and aids in family counseling.

FHM2 should be considered in patients with stroke-like presentations, particularly when accompanied by seizures or encephalopathy and in the context of a suggestive family history. Radiological findings may resemble ischemic stroke, underscoring the importance of comprehensive evaluation. This case highlights the need for increased awareness of FHM2 as a rare but important stroke mimic, and further studies are warranted to clarify optimal therapeutic strategies.

## Introduction

Migraine is one of the most prevalent neurological disorders, affecting approximately 15% of the global population, and represents a major cause of disability worldwide [1]. It is characterized by recurrent episodes of moderate-to-severe headache, frequently unilateral, and accompanied by associated symptoms including nausea, vomiting, photophobia, and phonophobia. In up to one-third of patients, migraine attacks are preceded or accompanied by aura symptoms, consisting of fully reversible focal neurological disturbances such as visual, sensory, speech, or language abnormalities [2]. While migraine with aura is common, hemiplegic migraine (HM) is a rare and distinctive subtype in which the aura phase includes transient motor weakness, a feature that may closely mimic acute ischemic stroke and other neurological emergencies [3].

HM is further classified into sporadic HM (SHM) and familial HM (FHM). FHM is inherited in an autosomal dominant manner and is genetically heterogeneous, associated with mutations in the CACNA1A, ATP1A2, and SCN1A genes [4-6]. These genes encode ion channel and transporter proteins that regulate neuronal excitability, and pathogenic variants lead to abnormal synaptic transmission and cortical spreading depression, the pathophysiological correlate of migraine aura [7,8].

FHM type 2 (FHM2) is specifically caused by mutations in ATP1A2, which encodes the α2-subunit of the astrocytic Na⁺/K⁺-ATPase pump. Dysfunction of this pump impairs potassium and glutamate clearance in the synaptic cleft, resulting in cortical hyperexcitability and susceptibility to spreading depolarizations [9,10]. Clinically, FHM2 can manifest with transient hemiparesis, sensory loss, aphasia, visual aura, epileptic seizures, altered level of consciousness, and, in severe cases, encephalopathy [11,12]. The overlap of these symptoms with acute ischemic stroke, autoimmune encephalitis, and infectious meningoencephalitis poses a significant diagnostic challenge, particularly in the emergency setting [13].

Accurate diagnosis of FHM is essential, as misdiagnosis may result in inappropriate or harmful interventions, such as administration of intravenous thrombolysis, mechanical thrombectomy, or prolonged courses of immunotherapy [14,15]. Neuroimaging findings may also contribute to diagnostic uncertainty. Although most patients have normal imaging, some cases of FHM2 have shown reversible cortical swelling, diffusion restriction, and even apparent large-vessel occlusion due to vasospasm, closely simulating stroke [16,17]. Given these complexities, genetic testing plays a pivotal role in confirming the diagnosis, guiding management, and facilitating family counseling [18,19].

Therapeutic strategies for FHM remain challenging due to the rarity of the condition and the lack of randomized controlled trials. Conventional migraine-specific therapies such as triptans and ergot derivatives are generally contraindicated because of their vasoconstrictive properties and potential risk of cerebral ischemia. Preventive agents reported to have benefits include calcium channel blockers such as verapamil, flunarizine, and nimodipine, as well as sodium channel stabilizers such as lamotrigine [13,14]. Acetazolamide has also been used anecdotally, particularly in genetically confirmed cases with frequent attacks [7,12]. Management is therefore largely individualized, focusing on avoidance of known triggers, judicious use of prophylactic therapies, and close monitoring in acute presentations where stroke and other secondary causes must be carefully excluded.

Here, we report a case of a genetically confirmed FHM2 presenting with prolonged hemiplegia, seizures, and encephalopathy, initially misinterpreted as an acute ischemic stroke. This case highlights the diagnostic pitfalls, neuroimaging findings, and therapeutic dilemmas associated with this rare disorder.

## Case presentation

A 33-year-old right-handed Caucasian female patient with no significant past medical history presented to the emergency department with a two-day history of severe, throbbing left-sided headache associated with dizziness, photophobia, phonophobia, vomiting, and diffuse abdominal pain. She also described progressive left-sided weakness affecting both upper and lower limbs, as well as new-onset slurred speech for 24 hours. There was no preceding fever, recent infection, or head trauma.

On arrival, her vital signs were stable, though she appeared distressed and photophobic. Neurological examination revealed left-sided lower motor neuron facial weakness, expressive dysphasia, pronator drift of the left arm, and Medical Research Council (MRC) grade 2/5 weakness in both the left upper and lower limbs. An extensor plantar reflex was noted on the left side. Sensory examination was limited due to patient cooperation.

The initial clinical impression was of an acute right middle cerebral artery (MCA) ischemic stroke. An urgent non-contrast CT of the brain demonstrated no evidence of intracranial hemorrhage or territorial infarction. In light of the acute presentation with hemiparesis, the patient was commenced on aspirin 300 mg orally for the presumed ischemic stroke.

However, within hours of admission, she developed generalized tonic-clonic seizures followed by a significant drop in consciousness (Glasgow Coma Scale (GCS) score 6/15), necessitating emergency intubation and admission to the intensive care unit (ICU). She was initiated on intravenous levetiracetam for seizure control and given empiric intravenous acyclovir for possible viral encephalitis. Her temperature subsequently rose to 39.5°C. After 24 hours, she was extubated, and her GCS improved to 13/15, though dense left hemiplegia and dysphasia persisted. A repeated neurological examination confirmed MRC grade 2-3/5 weakness in the left upper and lower limbs, persistent left facial palsy, and impaired fluency with preserved comprehension.

**Figure 1:**
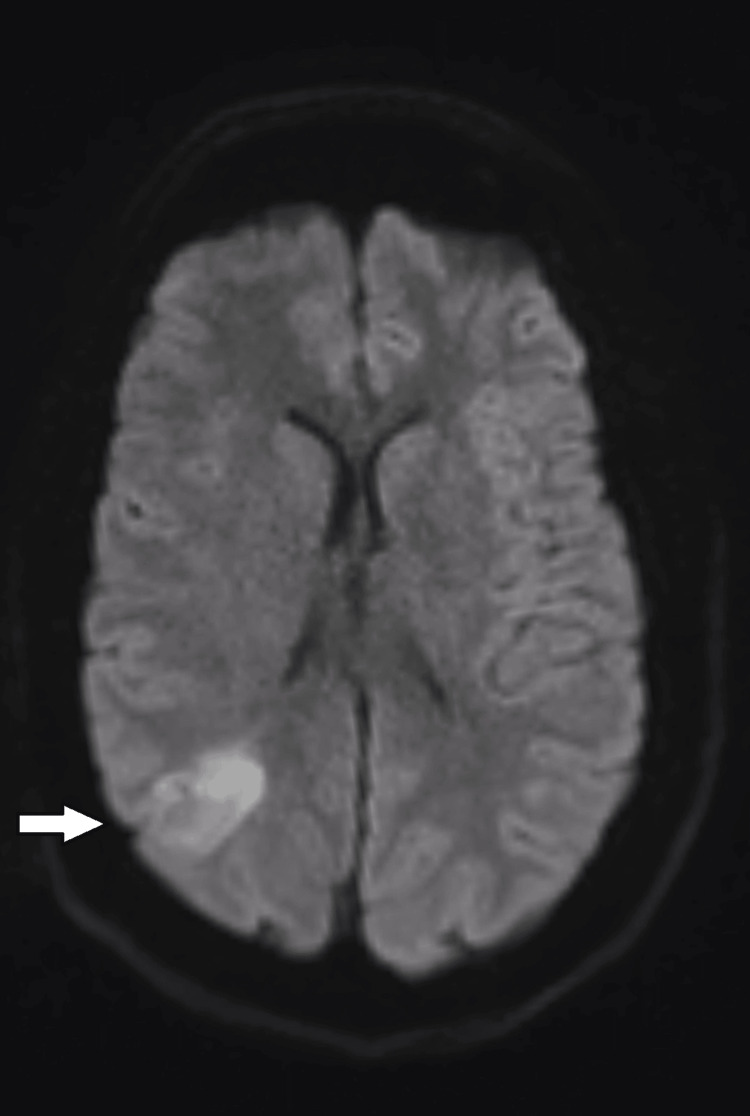
Axial DWI MRI of the brain Axial DWI MRI demonstrating a focal area of cortical diffusion restriction in the right parietal lobe (white arrow), extending into the adjacent subcortical white matter. The lesion appears hyperintense relative to the surrounding parenchyma, without associated midline shift or mass effect. Findings are in keeping with acute cortical involvement, initially suggestive of ischemia but subsequently attributed to familial hemiplegic migraine. MRI: magnetic resonance imaging; DWI: diffusion-weighted imaging

**Figure 2:**
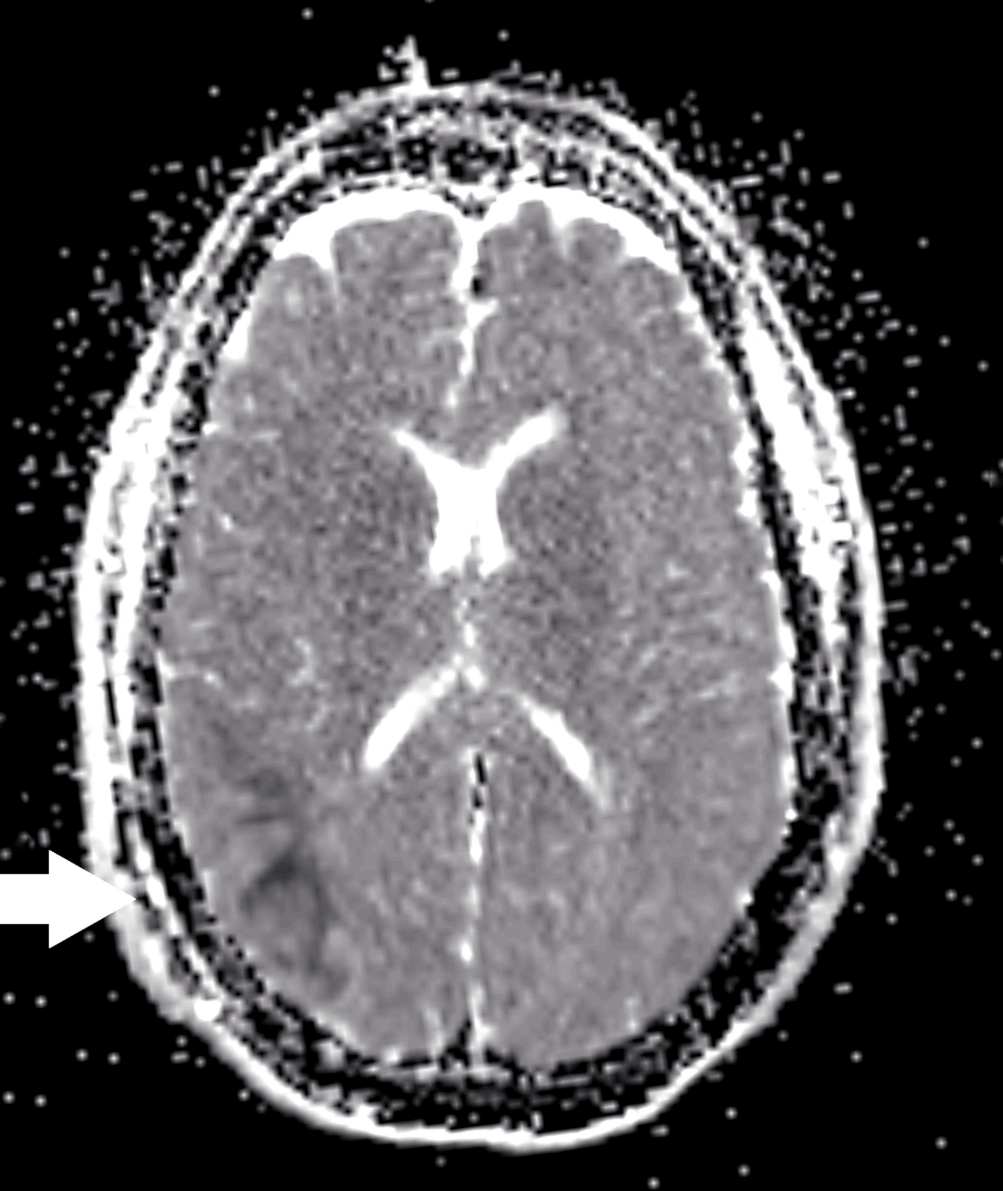
Axial ADC map of the brain MRI Axial ADC map corresponding to the DWI sequence, demonstrating reduced signal intensity in the right parietal cortex and adjacent subcortical white matter (white arrow). This finding correlates with the area of restricted diffusion seen on DWI, consistent with acute cortical involvement. ADC: apparent diffusion coefficient; MRI: magnetic resonance imaging; DWI: diffusion-weighted imaging

**Figure 3:**
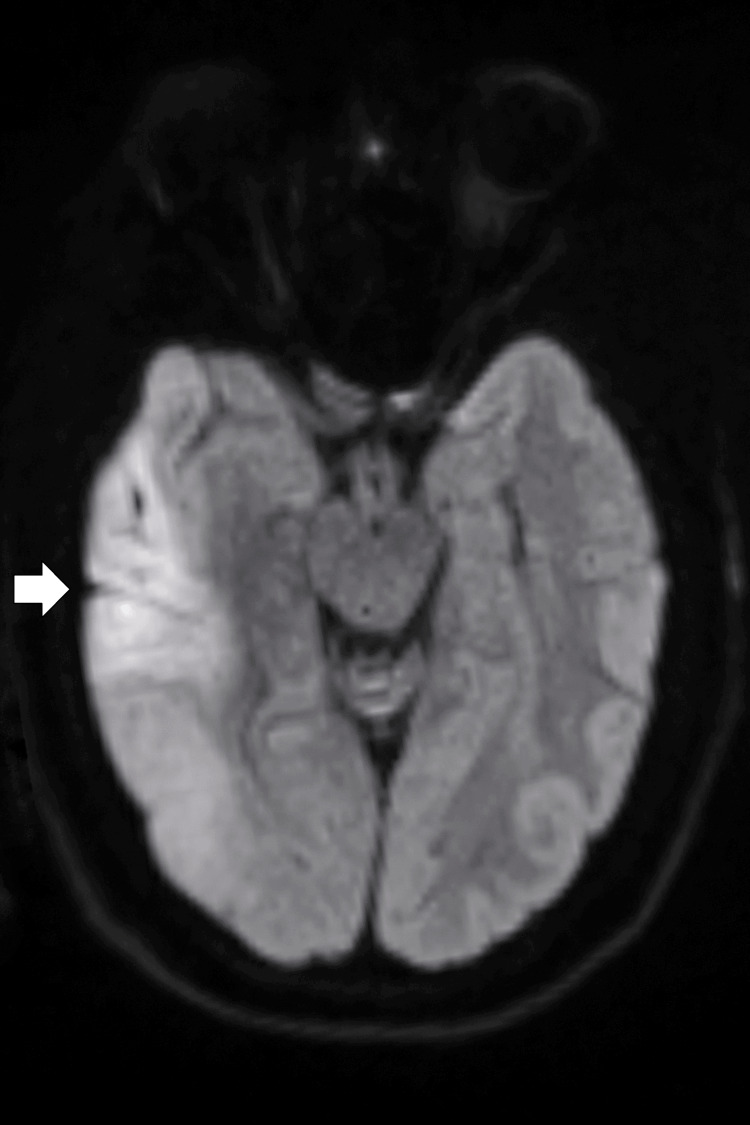
Axial DWI MRI of the brain Axial DWI MRI demonstrating a focal area of cortical diffusion restriction in the right temporal lobe (white arrow), extending into the adjacent subcortical white matter. The lesion appears hyperintense relative to the surrounding parenchyma, without associated midline shift or mass effect. Findings are in keeping with acute cortical involvement, initially suggestive of ischemia but subsequently attributed to familial hemiplegic migraine. MRI: magnetic resonance imaging; DWI: diffusion-weighted imaging

**Figure 4:**
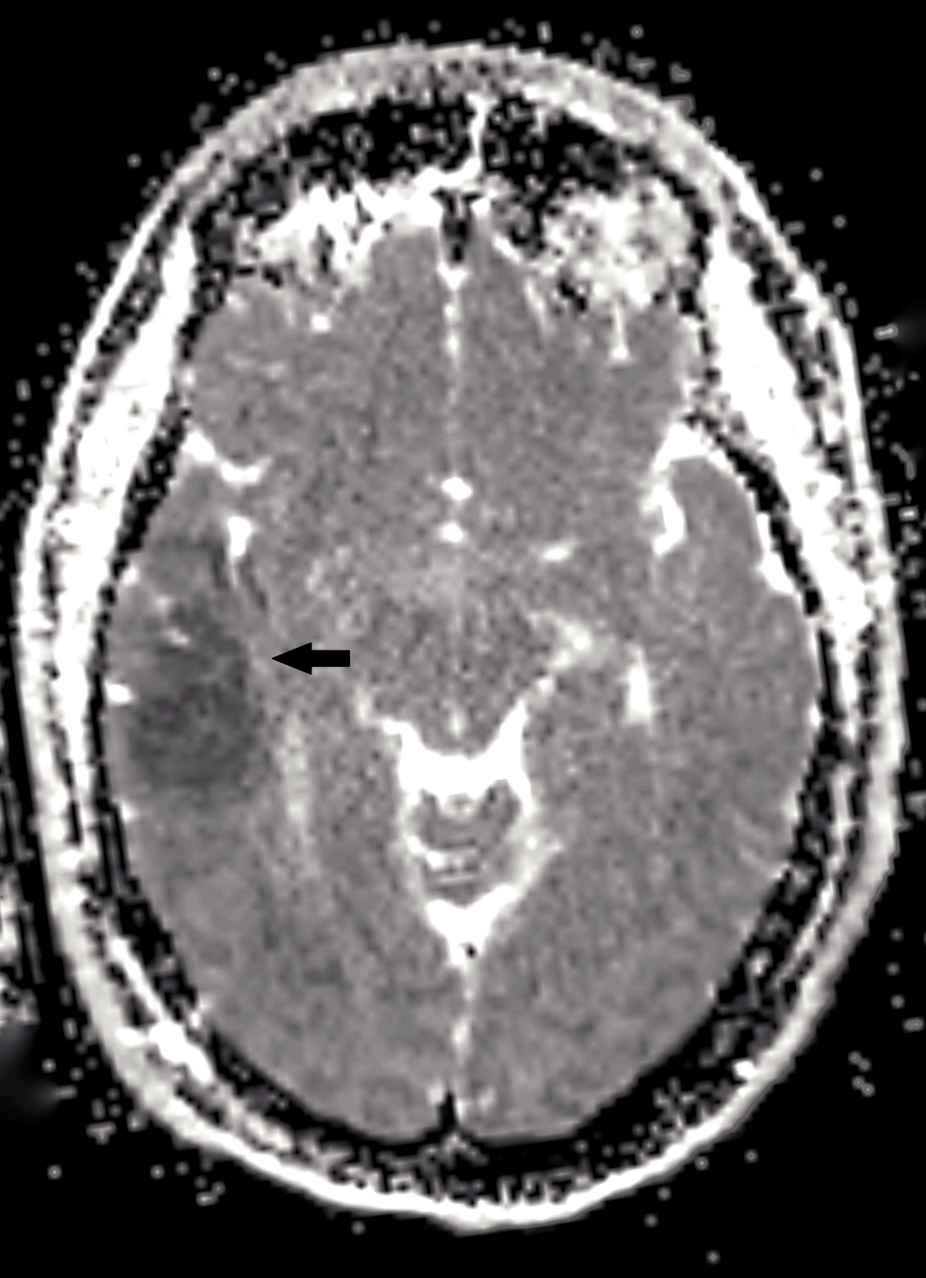
Axial ADC map of the brain MRI Axial ADC map corresponding to the DWI sequence, demonstrating reduced signal intensity in the right temporal cortex (black arrow). This finding correlates with the area of restricted diffusion seen on DWI, consistent with acute cortical involvement. ADC: apparent diffusion coefficient; MRI: magnetic resonance imaging; DWI: diffusion-weighted imaging

**Figure 5:**
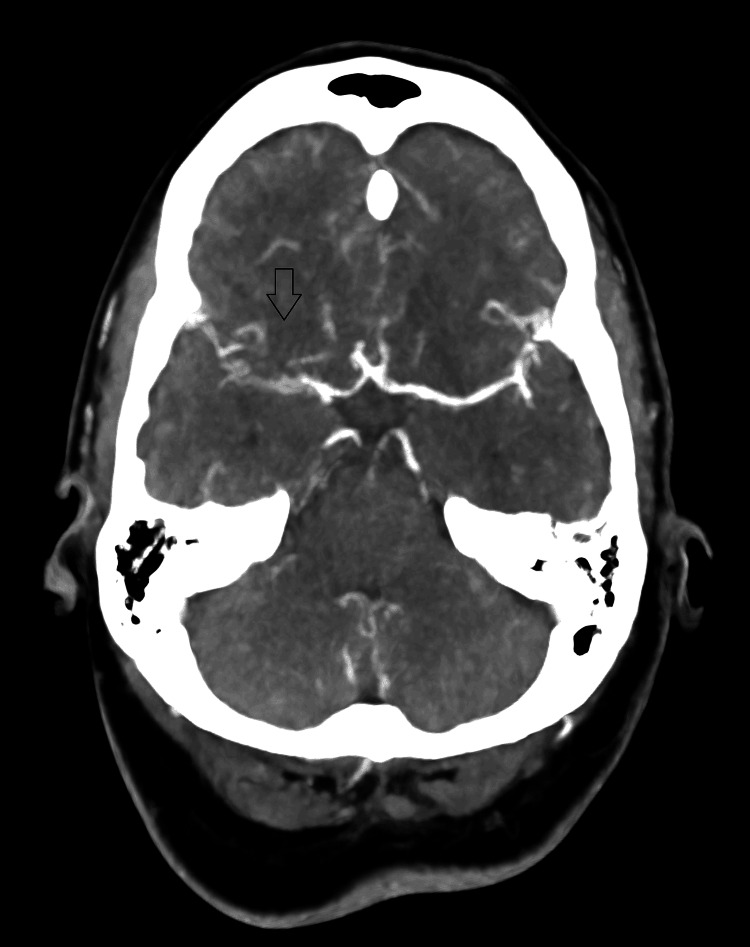
Axial CT angiography of the brain Axial slice from a CT angiography of the brain demonstrating reduced opacification of the right MCA M1 segment (black arrow) compared to the contralateral side. CT: computed tomography; MCA: middle cerebral artery

**Figure 6:**
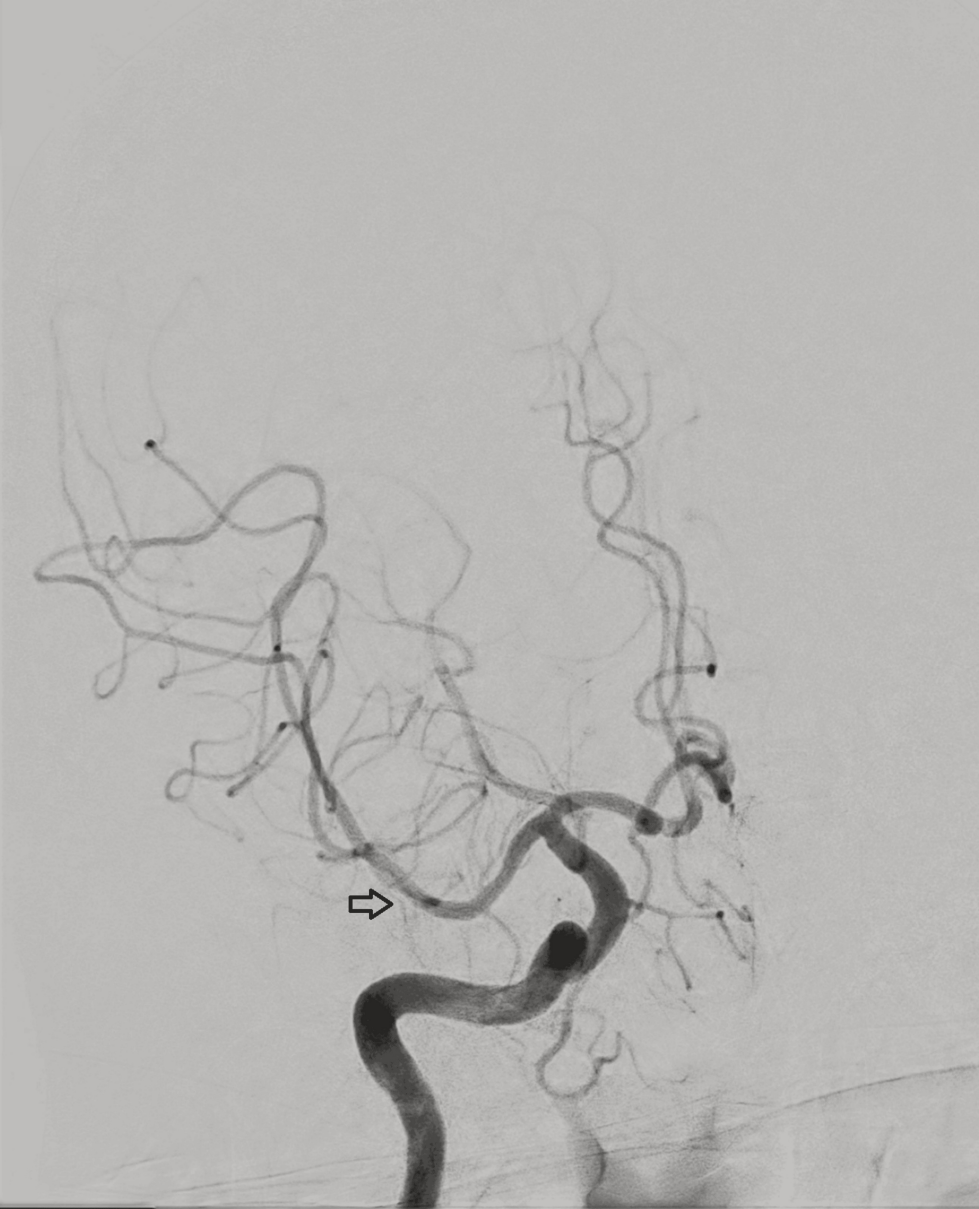
DSA of the right internal carotid artery DSA of the right internal carotid artery showing normal opacification of the MCA and its branches. A focal segmental narrowing in a distal MCA cortical branch (black arrow) is noted, suggestive of vasospasm, with no evidence of large vessel occlusion, thrombus, or aneurysm. DSA: digital subtraction angiography; MCA: middle cerebral artery

**Figure 7:**
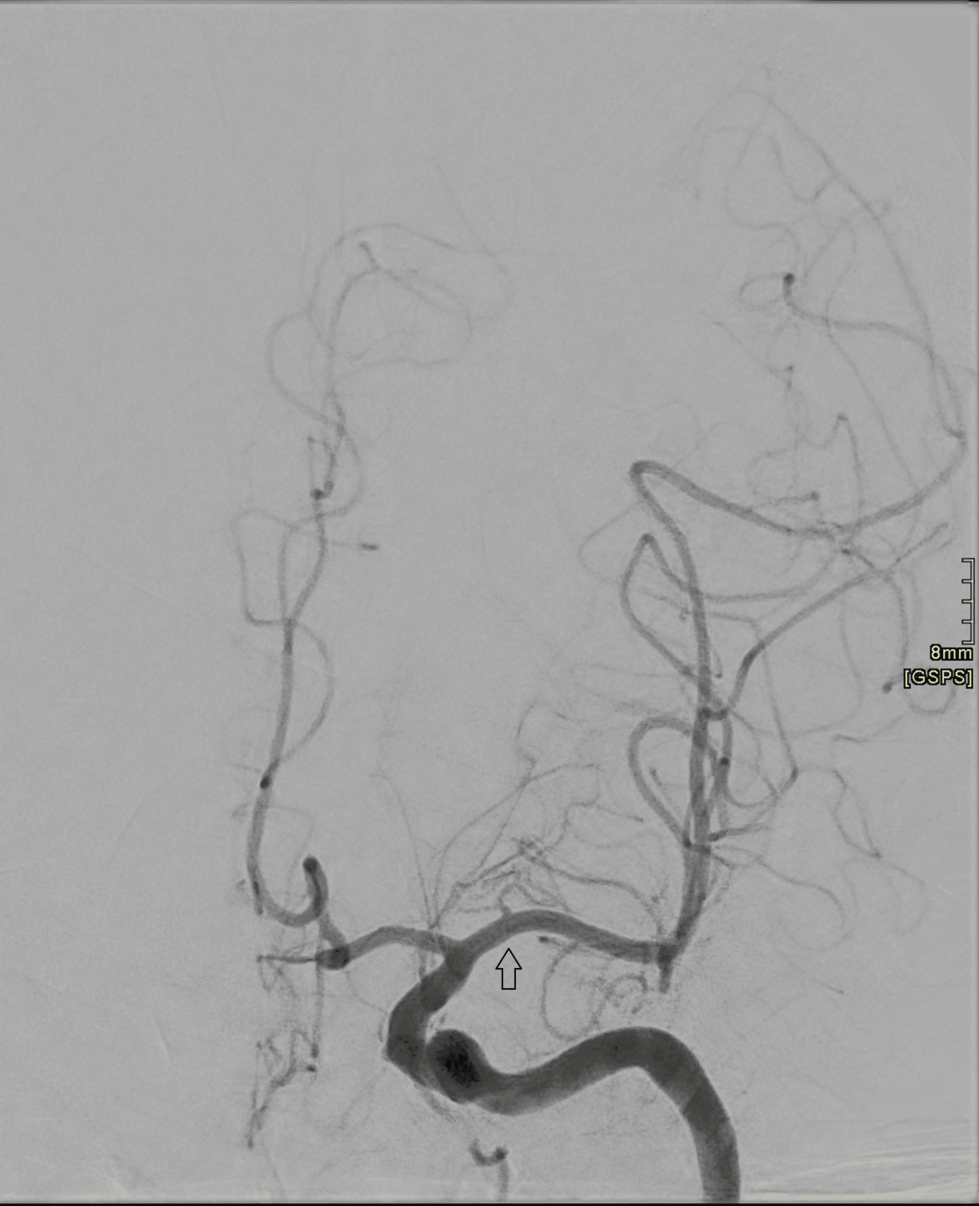
DSA of the left internal carotid artery DSA of the left internal carotid artery showing normal opacification of the MCA and its branches (black arrow). There is no evidence of segmental narrowing (in comparison with the right MCA in Figure 6), large vessel occlusion, thrombus, or aneurysm. DSA: digital subtraction angiography; MCA: middle cerebral artery

MRI of the brain (Figures 1-4) revealed cortical diffusion restriction involving the right parietal and temporal cortices, initially concerning for acute ischemia. However, the pattern was considered atypical for a single vascular territory infarct. Lumbar puncture showed clear cerebrospinal fluid (CSF) with normal protein and glucose and no evidence of pleocytosis or viral DNA. Autoimmune and paraneoplastic antibody screens, as well as an extensive infectious workup, were negative. CT angiography of the cerebral vessels (Figure 5) demonstrated an apparent right MCA (M1 segment) cut-off. This was initially interpreted as an acute thromboembolic event. Further discussion with the interventional radiology team clarified that the picture fits vascular spasm rather than a thromboembolic occlusion. Digital subtraction angiography (DSA; Figures 6, 7) was subsequently performed, which confirmed the patency of the right MCA; however, there was evidence of reduced diameter consistent with vasospasm compared to the left MCA.

Electroencephalography demonstrated low-amplitude background activity with generalized irregular 1-3 Hz delta slow waves, consistent with severe diffuse encephalopathy, but no epileptiform discharges.

A detailed collateral history revealed that the patient’s mother had a history of recurrent transient episodes of headache with unilateral weakness, previously labelled as transient ischemic attacks. This prompted further evaluation for hereditary migraine syndromes. The patient was commenced on oral nimodipine 60 mg every four hours as a preventive measure, with gradual improvement in her expressive dysphasia and level of consciousness over the subsequent days. Headache severity also decreased, and no further seizures were reported. However, residual left-sided weakness persisted at the time of discharge, with MRC grade 3/5 power in the left upper limb and 4-/5 in the left lower limb.

Genetic testing with whole-exome sequencing (WES) later confirmed a heterozygous mutation in the ATP1A2 gene, establishing the diagnosis of FHM2. The patient was referred for neurorehabilitation and outpatient neurology follow-up, with family genetic counseling arranged.

## Discussion

The clinical presentation in this patient, with acute hemiparesis, dysphasia, seizures, and encephalopathy, underscores the substantial overlap between FHM2 and acute ischemic stroke. Similar to Russell and Ducros [3], who reported frequent misdiagnosis of FHM as stroke or epilepsy, we observed that the initial presentation triggered stroke protocols. The recurrent episodes described by the patient’s mother mirror findings by Pelzer et al. [9], where family history played a pivotal role in distinguishing FHM from sporadic cerebrovascular or epileptic disorders. Consistent with Sutherland et al. [14], patients were initially misdiagnosed, highlighting the importance of early recognition to prevent inappropriate interventions.

Neuroimaging remains challenging in FHM. While non-contrast CT is often unremarkable, MRI may reveal cortical diffusion restriction resembling ischemic infarction [7,8]. In our case, diffusion restriction in the right parietal and temporal cortices was observed, echoing Tee et al. [8], who reported transient cortical diffusion changes in FHM patients that resolved without permanent infarction. CT angiography findings suggesting M1 segment occlusion were ultimately interpreted as vasospasm, consistent with Chabriat et al. [15], who documented transient perfusion abnormalities in FHM2 that mimic arterial occlusion. These parallels reinforce the utility of catheter angiography to distinguish true thromboembolic events from FHM-associated transient vascular changes.

Electroencephalography in our patient showed diffuse slowing indicative of severe encephalopathy. This aligns with prior observations by Costa et al. [17], where ATP1A2 mutations were associated with cortical dysfunction and seizures during prolonged attacks. Similarly, Tottene et al. [16] described enhanced cortical excitability underlying both hemiplegic symptoms and epileptiform activity in animal models.

Initial acute stroke management with aspirin was appropriate. Once FHM2 was confirmed, targeted therapy with nimodipine led to clinical improvement, consistent with previous reports by Dannenberg et al. and Sutherland et al. [13,14], who observed a reduction in attack severity with calcium channel blockers. The seizure management with levetiracetam also reflects established practice for ATP1A2-related epilepsy [17]. Our experience confirms that early recognition of FHM2 permits avoidance of contraindicated therapies, such as triptans or ergot derivatives, and allows for prophylactic strategies tailored to attack frequency and severity.

The patient’s recovery mirrors prior reports that while many FHM2 patients regain baseline function, prolonged attacks can leave residual deficits, particularly hemiparesis or cognitive impairment [19]. Hu et al. demonstrated that repeated episodes may contribute to cortical atrophy or white matter hyperintensities, reinforcing the importance of longitudinal monitoring [19]. Genetic counseling remains critical, as underscored by Jen et al. [18], both for family screening and anticipatory management.

## Conclusions

This case highlights FHM2 as a rare but important stroke mimic in young patients presenting with acute hemiparesis, dysphasia, seizures, and encephalopathy. The clinical overlap with acute ischemic stroke or encephalitis can lead to misdiagnosis and inappropriate interventions if clinicians are unaware of its hallmark features. This case underscores the critical value of a detailed family history, multimodal imaging, electroencephalography, and genetic testing in establishing an accurate diagnosis. Early recognition allows targeted therapy with calcium channel blockers and antiepileptics, which can be helpful and prevent inappropriate interventions such as thrombolysis or vasoconstrictive migraine medications, and facilitates long-term monitoring to reduce residual neurological deficits. Genetic confirmation informs family counselling and anticipatory management. Ultimately, an interdisciplinary approach involving neurology, radiology, and genetics optimizes patient safety and outcomes. Awareness of FHM2 among clinicians is essential to reduce diagnostic delays and ensure timely, effective care.
